# B Cell Signature during Inactive Systemic Lupus Is Heterogeneous: Toward a Biological Dissection of Lupus

**DOI:** 10.1371/journal.pone.0023900

**Published:** 2011-08-23

**Authors:** Jean-Claude Garaud, Jean-Nicolas Schickel, Gilles Blaison, Anne-Marie Knapp, Doulaye Dembele, Julie Ruer-Laventie, Anne-Sophie Korganow, Thierry Martin, Pauline Soulas-Sprauel, Jean-Louis Pasquali

**Affiliations:** 1 CNRS UPR 9021, Institut de Biologie Moléculaire et Cellulaire, Strasbourg, France; 2 Hôpital civil de Colmar, Colmar, France; 3 Institut de Génétique et de Biologie Moléculaire et Cellulaire, Illkirch-Graffenstaden, France; 4 Hôpitaux Universitaires de Strasbourg, Strasbourg, France; 5 Université de Strasbourg, Strasbourg, France; Beth Israel Deaconess Medical Center, United States of America

## Abstract

Systemic lupus erythematosous (SLE) is an autoimmune disease with an important clinical and biological heterogeneity. B lymphocytes appear central to the development of SLE which is characterized by the production of a large variety of autoantibodies and hypergammaglobulinemia. In mice, immature B cells from spontaneous lupus prone animals are able to produce autoantibodies when transferred into immunodeficient mice, strongly suggesting the existence of intrinsic B cell defects during lupus. In order to approach these defects in humans, we compared the peripheral B cell transcriptomas of quiescent lupus patients to normal B cell transcriptomas. When the statistical analysis is performed on the entire group of patients, the differences between patients and controls appear quite weak with only 14 mRNA genes having a false discovery rate ranging between 11 and 17%, with 6 underexpressed genes (*PMEPA1, TLR10, TRAF3IP2, LDOC1L, CD1C and EGR1*). However, unforced hierarchical clustering of the microarrays reveals a subgroup of lupus patients distinct from both the controls and the other lupus patients. This subgroup has no detectable clinical or immunological phenotypic peculiarity compared to the other patients, but is characterized by 1/an IL-4 signature and 2/the abnormal expression of a large set of genes with an extremely low false discovery rate, mainly pointing to the biological function of the endoplasmic reticulum, and more precisely to genes implicated in the Unfolded Protein Response, suggesting that B cells entered an incomplete BLIMP1 dependent plasmacytic differentiation which was undetectable by immunophenotyping. Thus, this microarray analysis of B cells during quiescent lupus suggests that, despite a similar lupus phenotype, different biological roads can lead to human lupus.

## Introduction

Systemic lupus erythematosous (SLE) is an autoimmune disease which is clinically and biologically characterized by a wide spectrum of signs variable from one patient to another. Indeed, the diagnosis of SLE mainly relies on the association of clinical and biological symptoms, some of which being validated as diagnostic criteria [1]. Not only different organs can be affected in groups of patients with SLE, but also the immunological hallmark of the disease, the autoantibodies, are diversely expressed with the exception of antinuclear antibodies which are quasi-constant in patients. This phenotypic heterogeneity of SLE patients may reflect different genetic contributions (i.e. various combinations of susceptibility genes) and/or different environmental factors which could lead to diverse immunopathological consequences. Among the many immune cell types which have been implicated in this heterogeneous disease, B lymphocytes appear central to the development of lupus and deserve further attention because: 1/they produce the autoantibodies, 2/they are activated during the disease, 3/they are responsible for the frequent hypergammaglobulinemia, and 4/they could present some intrinsic defects responsible for lupus traits and currently unknown. Indeed, in the spontaneous lupus prone mice NZB/W F1, it was shown that immature B cells from the parental lines NZB and NZW, when transferred to immunodeficient mice, produced hypergammaglobulinemia and antiDNA antibodies [2]. In the recent years, the B cell phenotype was extensively studied during lupus leading to the dissection of quantitative abnormalities of B cell subpopulations like naïve B cells, CD5 B cells, transitional B cells, memory and plasma B cells based on the expression of various membrane markers [3]–[8]. Some of these B cell abnormalities correlate with lupus activity and could reflect the extrinsic influence of various factors, like type I Interferons and/or BAFF, on the B cell subpopulations [9]–[11]. In an effort to track down putative intrinsic B cell defects during SLE, we analysed the transcriptomas of purifed B cells from inactive patients without immunosuppressive treatment, and compared the SLE B cell gene expression to healthy individual B cell transcriptomas. This approach, using purifed B lymphocytes instead of a mixture of peripheral mononuclear cells and non hypothesis driven large scale microarrays, should be able to point out the implication of some biological pathways, and to define such intrinsic B cell defects. The overall statistical analysis of the differential gene expression (17 patients versus 9 controls) identified a very low number of genes with an acceptably low false discovery rate (FDR) showing that gene expressions were quite similar between quiescent lupus B cells and controls. However, a subgroup of patients was clearly distinct from the others and from the controls, with differentially expressed genes mainly implicated in plasmacytic differentiation and confirming at the B cell level the heterogeneity of the pathways leading to lupus.

## Materials and Methods

### Patients

17 patients (15 females and 2 males) with the diagnosis of SLE were selected for this study after they gave their informed consent. The SLE diagnosis was based on the presence of at least 4 criterias among those defined by the American College of Rheumatology. The lupus was inactive in these patients for more than 6 months, with a Systemic Lupus Erythematosous Disease Activity Index (SLEDAI) score less than 4 [12], and they did not receive any immunosuppressive drug. If they needed steroids, the patients were not treated with more than 10 mg of prednisone per day (4 patients). 10 patients were treated with hydroxychoroquine. The clinical characteristics of the patients are presented in Table 1. The 10 control subjects were healthy individuals, (8 females and 2 males) ageing from 23 to 53 years, with no personal nor familial history of autoimmune disease. 17 patients (15 females and 2 males) with the diagnosis of SLE were selected for this study after they gave their written informed consent. This study was approved by the ethic comity of the Hôpitaux Universitaires de Strasbourg.

**Table 1:** Clinical features, and disease activity index at the time of the study.

Patient n°	Age	Sex	Duration of disease (years)	SLEDAI	IgG levels (g/l)	ANAstitle	Anti-dsDNA	Steroids	Chloroquine
1	36	F	3	0	8.49	1/160	−	−	−
2	59	F	20	3	8.15	1/1280	+	−	+
3	38	F	18	0	8.29	1/1280	−	−	+
4	37	F	9	2	7.3	1/640	+	+	+
5	36	F	8	4	7.22	1/1280	+	−	−
6	36	F	9	0	10.2	1/160	−	−	−
7	41	M	9	0	11.2	1/640	−	−	−
8	55	F	15	4	13.8	1/320	−	−	−
9	47	F	9	0	15.9	1/320	−	−	−
10	36	F	12	4	7.68	1/1280	+	−	−
11	37	F	8	2	9.47	1/1280	+	+	+
12	53	F	19	0	9.76	1/1280	−	+	+
13	30	F	2	0	7.8	1/160	−	−	+
14	41	F	7	0	11.9	1/1280	−	+	+
15	23	M	8	4	7.3	1/640	+	−	+
16	37	F	18	4	8.07	1/1280	+	−	+
17	50	F	26	0	10.5	1/640	−	−	+

Disease and treatment were stable for at least 6 months. SLEDAI: Systemic Lupus erythematosus disease activity index; IgG normal range: 7–14 g/l, ANA: antinuclear antibodies

### B lymphocyte preparation and RNA purification

Peripheral blood was drawn into heparin-containing sterile tubes and peripheral blood mononuclear cells were prepared by Ficoll (Amersham) density gradient centrifugation for immediate use. B cells were labeled with a biotin anti-CD19 monoclonal antibody (HIB19 clone, Pharmingen) at 4°C and revealed by phycoerythrin-labelled streptavidin (Biomeda) before immediate B cell sorting with high speed cell sorter (FACS Diva, Beckton-Dickinson). Total RNAs from the sorted B cells were extracted using TRIzol reagent (Invitrogen) according to the manufacturer's instructions. They were then precipitated in Glycogen (Invitrogen) and suspended in DNAase-free and RNAase-free water (Gibco). The quality of the RNA preparations was always checked with RNAlabChip (Agilent) before any further step. Good quality RNA preparations (approximately 50 ng per preparation) were amplified using the Affymetrix 2 cycle cDNA synthesis kit. In order to reduce the variability of these preparations, one control B cells and generally 2 patients' B cells preparations were treated simultaneously.

### Gene micro arrays preparations and GeneChip analysis

**Figure 1:**
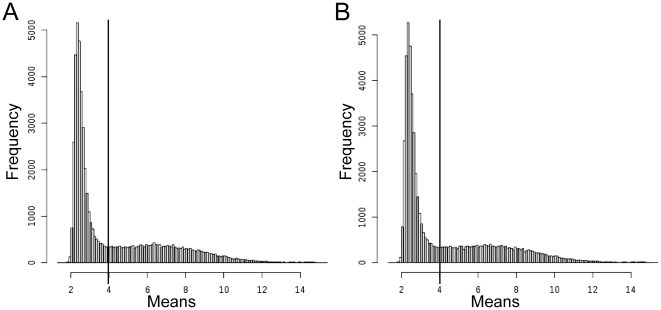
Histogram distribution of GCRMA expressions values. Genes with expression values below 4 were considered unexpressed in B cells both in patients (A) and in control (B).

cRNAs were synthesized, biotin-labelled and hybridized to the Affymetrix GeneChip human genome U133 plus 2.0 (with probe sets representing 38,572 UniGene clusters) according to the manufacturer's instructions. After hybridization and washings, arrays were stained with PE-conjugated streptavidin (10 µg/ml) before scanning. Raw Affymetrix data (available at http://www.ncbi.nlm.nih.gov/projects/geo/query/acc.cgi?acc=GSE30153) were analyzed using R (R Development Core Team, 2008; The Comprehensive R Archive Network: http://cran.r-project.org/) and Bioconductor (Bioconductor: http://www.bioconductor.org/) softwares [13]. The quality of the 27 Affymetrix genechips and RNA was assessed using the Bioconductor AffyPLM and simpleaffy packages, with qc, AffyRNAdeg, fitPLM, image, RLE and NUSE functions: one control chip showing too many defects was left aside, thus further analysis were carried out on 9 control and 17 patient chips. For normalization and background correction, Raw values were pre-processed with RMA or GCRMA (library simpleAffy). As further analysis with RMA or GCRMA data gave similar results, only results with GCRMA expression values will be shown. According to the histogram distribution of GCRMA expression values (Fig. 1), we considered as unexpressed genes (both in patients and controls), genes with expression values below 4. Genes with values lower than this threshold were eliminated: of the 54,675 Affymetrix probe sets, only 18,271 (33%) correspond to genes expressed in B lymphocytes. Identification of differentially expressed genes and estimation of the False Discovery Rate (FDR, [14], [15]) were carried out using the Significance Analysis of Microarrays (SAM) algorithm available in the siggenes package [16].

### Data clustering

The dist and hclust functions of the simpleaffy library were used to build hierarchical clusterings of the data.

### QRT-PCR Analysis

cDNAs from total RNAs were prepared after patients and control B cell separations using the high capacity cDNA Reverse transcription Kit (Applied Biosystems). 10 ng of each cDNA was subjected to QRT-PCR using Applied Biosystems TaqMan assays (validated for each selected gene) on the ABI Prism 7000 instrument. The ΔΔCt provided the target gene expression value by comparison with a calibrator sample (Applied Biosystems). The patients and control samples and the calibrator were first normalized by the relative expression of the 18s.

### B Lymphocyte ligands and lupus

Zhu et al. in 2004 published an extensive analysis of mouse splenic B cell gene expression changes in response to in vitro stimulation with 33 ligands of B lymphocytes [[17], and Data available online: UCSD-Nature Signaling Gateway, Alliance for Cellular Signaling, AfCS Data Center, B-cell ligand screen, http://www.signaling-gateway.org/data/cgi-bin/table.cgi?cellabbr=BC]. In an attempt to find associations between human SLE and these ligands, we compared patterns of genes differentially expressed in B lymphocytes during SLE (our results in the subgroup of 5 patients) and in response to these ligands (Zhu's results). In order to identify homolog human and mouse genes, i.e gene with the same symbol name, clone identifiers are converted to gene symbol names. For Affymetrix probe sets the conversion to gene symbols is straightforward using the Affymetrix NetAffx Analysis Center (http://www.affymetrix.com/analysis/index.affx). In contrast the conversion of mouse clone ID to symbol names require queries to several data bases as the custom Agilent cDNA Microarray chip used in Zhu et al. publication was made up of clones from four libraries: RIKEN, NIA, Research Genetics, and Genome systems.The Representing Factor (http://www.nemates.com/uky/MA/progs/overlap_stats.html) and the Resampling statistical methods [18], and available online: http://www.resample.com/content/text/index.shtml] were used to compare the patterns of genes differentially expressed in human and mouse B lymphocytes. The identification of the biological pathways and of the ontology groups (biological processes and molecular functions) of selected list of genes differentially expressed in the 5 lupus patients subgroup was performed using the DAVID program with a Bonferroni correction for multiple testing (DAVID Bioinformatic Resources, NIAID, NIH), and the CYTOSCAPE program with the MiMI plugin [19].

## Results

The patients' characteristics are presented in Table 1. They were all considered of having an inactive phase of SLE with a variable disease duration (2 to 26 years). B cell purity was checked by FACS analysis of sorted CD19 positive cells ( more than 96%).

### Differentially expressed genes in lupus B cells compared to normal B cells

According to the MIAME recommendations, the data discussed in this publication have been deposited in NCBI's Gene Expression Omnibus and are accessible through GEO Series accession number GSE30153 (http://www.ncbi.nlm.nih.gov/projects/geo/query/acc.cgi?acc=GSE30153) as well as the full normalized and annotated results of the RMA analysis of the 2,327 genes with initial p values of less than 0.05 (Table S1). Then, the B cells transcriptional profiles originating from the 17 lupus patients compared to the 9 normal individuals were analysed using the SAM algorithm and multiple testing correction according to Benjamini et al [14], [15]. Using this stringent statistical analysis, and after removing the upregulated Ig genes from this short list, it appears that only a very small number of genes (14 out of the 18,271 which were expressed in B cells) are differentially expressed with a FDR ranging from 11 to 17% (Table 2). At first glance, these results indicate that, at the transcriptomal level, and during the inactive phases of the disease, lupus B cells are very similar to normal B cells. The differentially expressed genes were checked by real time qPCR only in a few patients (because of the availability of the cDNAs) and were confirmed to be up or downregulated during lupus. Among these genes, it is interesting to note that TRAF3IP2 (alias ACT1) is a negative regulator of B cell function, its absence leading to lymphoproliferation and autoantibody production [20], but ACT1 is also essential in IL-17 dependent signaling during autoimmune diseases [21], IL-17 being implicated during lupus physiopathology [22]. On the other hand, the low level of expression of CD1c mRNA could be related to the fact that CD1c is highly expressed on unswitched memory B cells or circulating counterpart of marginal zone B cells [23], this subpopulation being decreased during the inactive phase of lupus [24]. TLR10 has to date no defined agonist or function but is apparently functional with a distinct signaling pathway in B cells [25], [26].

**Table 2:** Genes over or underexpressed in lupus patients B cells (FDR from 11 to 17%) compared to control B cells.

Probes	Xfold(log2)	Unigene	Gene symbol	Function(NCBI)
1554474_a_a209708_at	1.93	Hs.6909	MOXD1	Catecholamine metabolism
201890_at209773_s_at	4.44	Hs.226390	RRM2	Oxidoreductase activity, implicated in DNA replication
202589_at1554696_s_at	3.79	Hs.592338	TYMS	DNA replication and repair
201543_s_at210790_s_at	1.97	Hs.499960	SAR1A	GTPase activity, intracellular protein transport
228486_at228485_s_at	2.32	Hs.573495	SLC44A1	Transmembrane transport
201923_at	1.98	Hs.83383	PRDX4	Antioxidant enzyme, regulatory role in the NF-kappaB pathway
203857_s_at	2.63	Hs.477352	PDIA5	Isomerase activity, protein folding
39249_at	1.40	Hs.234642	AQP3	Glycerol and water channel activity
222450_at	0.49	Hs.517155	PMEPA1	Androgen receptor signalling pathway
215411_s_at	0.71	Hs.654708	TRAF3IP2	Positive regulation of I-kappaB kinase/NF-kappaB cascade
223751_x_at	0.67	Hs.120551	TLR10	Innate immunity Potential Pam(3)CSK(4) receptor
205987_at	0.42	Hs.132448	CD1c	Presentation of primarily lipid/glycolipid antigens
223228_at	0.66	Hs.715637	LDOC1L	Unknown
227404_s_at201694_s_at	0.38	Hs.326035	EGR1	Transcriptional regulator

### A subset of SLE patients have a distinct gene expression profile

**Figure 2:**
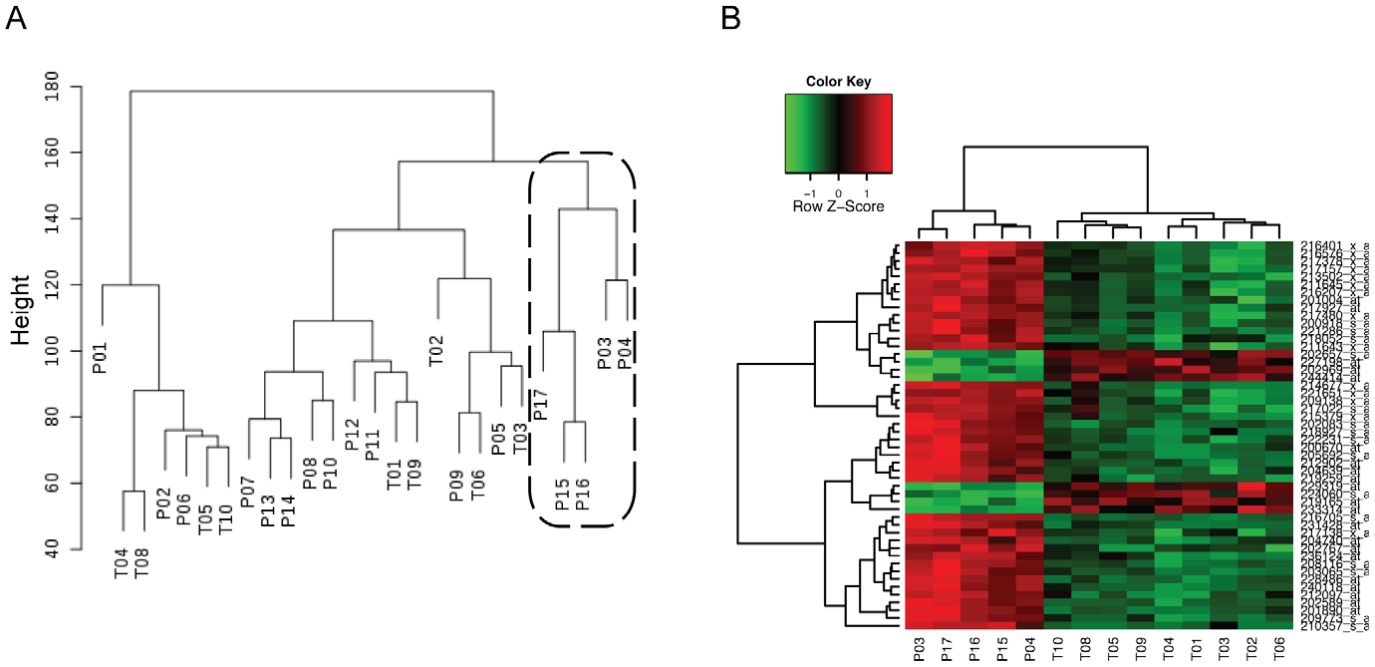
A subgroup of 5 patients stands out from the others. (A) Dendrogram obtained by unforced hierarchical clustering of the microarrays from the 17 patients and the 9 controls. A subgroup of 5 patients (surrounded by a dashed line) stands out from the others. (B) Heat-map of the 50 first differentially expressed genes in these 5 patients compared to controls after filtering the results for low signal. Over-expressed genes are shown in red and under-expressed are depicted in green.

**Figure 3:**
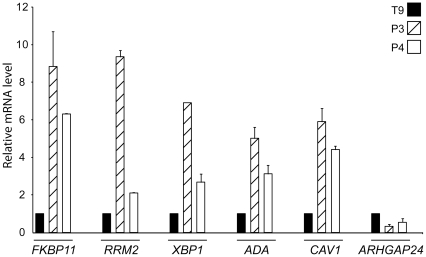
Quantitative RT-PCR of 6 selected genes in 2 patients and one control. *FKBP11, RRM2, XBP1, ADA, CAV1* and *ARHGAP24* expressions were determined by real time quantitative RT-PCR. Each sample was normalized to the endogenous control 18S.

Unforced hierarchical clustering of the patients and the controls was performed with the 18,271 genes expressed in the B cells. The figure 2A shows that the gene expressions of the patients and the controls were quite similar, confirming the previous statistical analysis. However, the same unforced hierarchical clustering identifies a subgroup of 5 patients with a distinct gene expression profile. The statistical analysis comparing the gene expression of these 5 patients with the controls and the other patients was indeed highly significant. Extremely low FDRs (less than 0.01) were associated with the differential expression of approximately 800 genes (Table S2, and Heat-map of the first 50 genes in Fig. 2B). Considering the availability of the mRNAs (which was the limiting factor), we only checked by real time qPCR the expression levels of 6 selected genes in 2 patients and one control (ADA, RRM2, CAV1, XBP1, ARHGAP24, FKBP11) and confirmed the microarray results (Fig. 3). Looking for the origin of this peculiar gene expression profiles in these 5 patients, we first tried to find differences in the clinical phenotype of the patients but we were unable to find such differences (gender, age of onset, disease duration, activity score, levels of serum Ig and anti nuclear antibodies, treatments at the time of sampling). Second, since the difference could originate from a distinct representation of the B cell subpopulations in these 5 patients, we checked the detailed cytofluorometric patterns (CD19, IgG, IgM, IgD, CD138, CD27, CD86) observed on B cells from both controls and patients. Differences were evidenced between the patient and the control groups [24], but we did not see any statistical difference between the 2 patients subgroups (12 versus 5): equivalent percentages of CD19/CD138 double positive cells, CD19/CD27 double positive cells, and CD19/CD86 double positive cells were found. Third, the differential gene expression could originate from B cell extrinsic or intrinsic properties pointing to original biological pathways in these 5 patients.

### Ligand signature?

Still focusing on the differential gene expression between the subgroup of patients and the controls, the results could represent intrinsic or extrinsic gene expression abnormalities or both. In order to approach the possible influence of extrinsic factors, we took advantage of the Signaling gateway data center which gives the results of an extensive analysis of microarrays performed on murine purified splenic B cells during in vitro stimulation with 33 different ligands [17]. Thus, we compared our list of in vivo differentially expressed human genes during SLE with differentially expressed murine genes under influence of these ligands. To be precise, we specifically compared the differentially expressed (1.5 fold change) murine genes after 4 h ligand stimulations with our list of highly differentially expressed genes originating from the subgroup of the 5 SLE patients( thereby named SLE list). Different steps were required: 1/only genes which were present on both murine and human microarrays were considered for this analysis, 2/we identified the different ligand regulated gene files, 3/among these files, we identified the murine genes which were also present in the SLE list (common lists of genes) and counted the number of genes varying in the same direction for each ligand, 4/then, for each ligand, we calculated the Representing Factor and the associated probability of finding an overlap set of genes [http://www.nemates.com/uky/MA/progs/overlap_stats.html]. This calculation leads to identify IL4 imprinting as the only significant signature in these 5 patients B cells: the common list of genes for IL4 contained 112 genes with 101 genes varying in the same direction. This was confirmed by another statistical method (Resampling, [18]). Type I Interferon (only 35 genes on the common list, with 30 moving in the same direction), BAFF (33 genes on the common list, with 25 moving in the same direction) and CD40L (84 genes on the common list, but 59 only varying in the same direction) did not reach statistical significance.

### Biological pathways in the subgroup of 5 patients

In order to analyse the biological significance of differentially expressed genes in these 5 patients compared to controls, different tools are available. We used the DAVID program to look for statistically represented biological pathways. If we enter the SLE list of genes into the DAVID program, it appears that one biological pathway is highly significantly overrepresented after a Bonferroni correction for multiple testing: the endoplasmic reticulum (p less than 8.8×10^−11^). Among these genes which point to the endoplasmic reticulum, a large set of genes participate to the Unfolded Protein Response. Many of these genes are controlled by the overexpression of BLIMP1, a master regulator of B cell terminal differentiation: DNAJC3, SEC61A, BIP, SSR4, PPIB, RPN1… This overexpression of BLIMP1 mRNAs is not related to EGR1 because the later is also down regulated in these 5 patients [27]. On the other hand, XBP1, whose mRNAs are also overexpressed, could be inactive since its specific target genes are not upregulated (SLC30A, ARHQ, OBF1). It is interesting to note that 1/IL4 is indeed able to induce XBP1, but not the IRE1 activation induced XBP1 splicing which is necessary to produce the active form of XBP1 [28], and not to induce BLIMP1, and 2/BI1 mRNAs (Bax Inhibitor 1) are increased in these B cells and BI1 is known to repress IRE1 activation [29].

At the level of gene interactions, using Cytoscape and the MiMI program, analysis indicates complex relationships between differentially expressed genes which can belong to distinct biological pathways. As an example, we can mention the complex network of possible interactions between FYN, whose mRNAs are down regulated in these patients, and 8 directly interacting gene products whose mRNAs are overexpressed in the same cells.

## Discussion

Based on two main considerations (the central role of B cells during SLE, and the possible intrinsic abnormalities of SLE B cells), we performed the transcriptomic analysis of purified B cells during non active phases of the disease. Such an analysis 1/should reduce the variability of the transcriptomas because of the purity of the analysed cells, and 2/should reduce the risk of focusing on gene expressions associated with lupus flares and their medical treatments. The interpretation of the microarrays is here limited to B cells, avoiding difficulties in data mining linked to heterogeneous populations of cells present in the peripheral blood mononuclear cells in unknown proportions [30]. Still the interpretation can be obscured by the presence of different B cell subpopulations in the human peripheral blood. Indeed, in a separate set of experiments starting with the same blood samples, we performed a detailed B cell immunophenotyping which showed some differences between control B cells and SLE B cells [24], some of which being potentially able to explain differentially expressed genes. Despite these differences, one of the main results of our study is the important similarity at the transcriptomic level between normal B cells and SLE B cells during non active phase of the disease. At a threshold close to 10% for the FDR (upper limit of reasonably acceptable risk for microarray analysis [31]), only 14 genes out of 18,271 appear differentially expressed. The biological significance of these differences could be diverse. For instance, the down expression of CD1c mRNAs could be related to the low percentage of the CD1c high unswitched memory B cells among total SLE B cells. On the other hand, the down expression of ACT1 (TRAF3IP2), could be linked to SLE because of the importance of this negative regulator on the B cell function [20]–[22]. At that stage, it is almost impossible to compare the results of the different SLE wide genome scans with our microarray results because of the very limited informations of the functionality of the different polymorphisms which were described. However, it is interesting to note that BLK (C8orf13) does not appear on our list of differentially expressed genes despite the B cell down expression of this kinase when its regulatory region expresses the “SLE” polymorphism [32]. Whether this polymorphism is present or not in our patients, or whether the downregulation of BLK only occurs during an active phase of the disease, remains to be determined.

The second main result of our data is related to the transcriptomic heterogeneity of the patients. The unforced hierarchical clustering of the patients and controls revealed a subgroup of 5 patients with a distinct pattern of mRNA expression in B cells leading to the identification of a set of genes with a high statistical significance. Looking for clinical or biological peculiarities in these 5 patients, we did not find any difference with the other 12 SLE patients. We also compared their B cell subpopulations patterns, but again did not find any difference. Thus, we are left with the possibility that the B cell signature of these 5 patients could be the result of either extrinsic or intrinsic B cell properties.

Looking for an extrinsic signature of the SLE B cell transcriptoma in these five patients, we found a significantly enriched expression of genes induced by Il4. However, this approach has several limits: 1/ligand induced gene expression in purified B cells could be different in mouse and human, although generally speaking these biological pathways are quite conserved, 2/for comparisons with our human gene list, we only considered mouse genes that were consistently modified 4 h after in vitro ligand stimulations, which could ignore some interesting early and late gene changes, 3/the analysis can be obscured by the frequent sharing of expression change patterns between different ligands (anti-Ig, CD40L, BAFF, IL-4, CpG, Type I Interferons, data not shown). Having in mind these limitations, it appears that B cells from these 5 inactive SLE patients have only one weak signature, although we did not find an increase of serum IL-4 level in these patients (data not shown). However, the serum level of IL-4, or the IL-4 production by peripheral blood mononuclear cells during lupus is not clear, with conflicting results maybe linked to the activation status of the patients [33]-[36]. It is interesting to note that the type I interferon signature which was reported during active SLE was not clearly detected during the inactive phase of the disease.

To look for intrinsic B cell defects in these 5 patients, we removed from the list of differentially expressed genes all those that were shown to be in vitro ligand regulated [17]. Analysis of the gene product interactions through the Cytoscape program gives some interesting clues. For instance, the Src family kinase FYN could be central to the disease: it is slightly down regulated in almost all the patients, and FYN deficiency in mice induces a tendency to produce anti-DNA antibodies and proteinuria through a non immunological mechanism [37]. FYN appears physically connected to 8 gene products whose mRNAs are overexpressed in the subgroup of 5 patients (SLAMF1, RICS, CSF2RB, CAV1, CDK5, CASP3, IL2RB, ATXN1 [38]–[42]) and could compete for FYN. The consequences on B cell biology of such a competition between the possible overexpressed proteins and the deficient target FYN are currently unknown. Looking for the origin of low FYN expression in these 5 patients, it is interesting to note that it is associated with the down expression of EGR1 which is known to control the FYN gene expression through an EGR1 binding site located in the promotor region of FYN [43]. Beside FYN, other genes coding for adhesion molecules like ICAM and CD44, can be regulated by EGR1 [44], [45] their mRNAs being down regulated in these patients as well.

**Figure 4:**
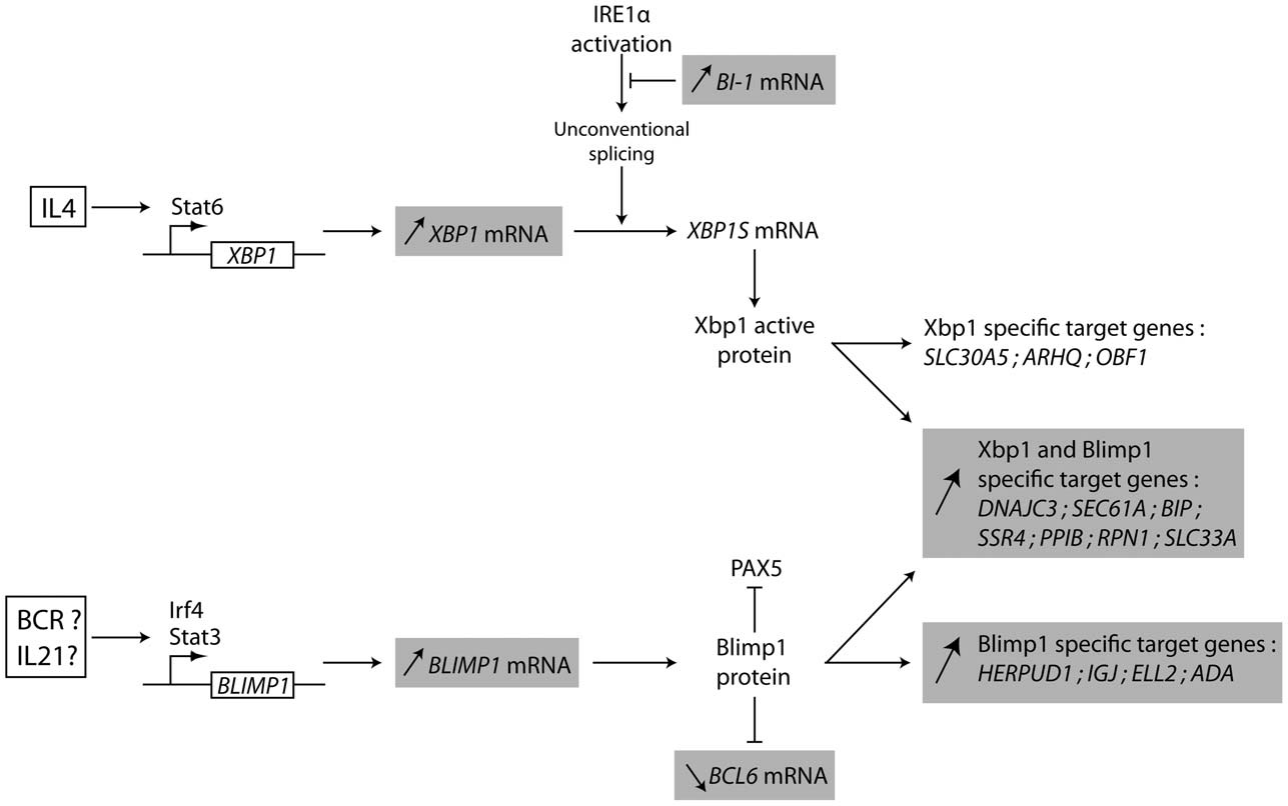
The observed significant variations of mRNAs in the B cells of the five patients are surrended and suggest a *BLIMP1* induced partial plasmacytic differentiation.

In order to find activation of biological pathways in the B cells of these 5 patients, we used the DAVID program. It identifies a large overrepresented set of genes which are deregulated during the plasmacytic differentiation of B cells as well as during the Unfolded Protein Response in different cell types. The fine analysis of the deregulated genes in these B cells suggests that B cells underwent Blimp1 induced partial plasmacytic differentiation, but without further terminal plasma cell differentiation (Fig. 4): BLIMP1 and XBP1 mRNAs are both overexpressed, but XBP1 could be inactive because 1/HERPUD1, ADA and ELL2 ( all being target genes for BLIMP1, but not for XBP1) mRNAs are increased, 2/On the contrary, specific target genes for XBP1 (SLC30A5, ARHQ) are not deregulated, which is consistent with an IL-4 influence [28], [46]. The precise stimulus that induces BLIMP1 over expression, but not XBP1 splicing, is not clear but if IL-21 is an obvious candidate, others are still possible alone or in combination: BCR, Calcineurin/NFAT, CD40/NFkB [47]. On the other hand, a new polymorphism associated with SLE was recently described in the vicinity of the BLIMP1 gene locus, suggesting an intrinsic property of B cells bearing this variant [48]. Thus, it seems that, in these patients, B cells are in a stage of intermediate differentiation, maybe arrested at a step before IRE1 induced XBP1 unconventional splicing which is necessary to produce the active form of the protein required for the full plasmacytic differentiation [49], [50]. Consistent with this hypothesis, the serum levels of IgG were not different in these 5 patients compared to the 12 others, but 20 out of the 32 genes of the plasmablast signature (module M1.1, [51]) are present in our list.

Such a possible stage opens new questions: 1/is this developmental arrest an intrinsic (constitutive) abnormality of B cells in these patients, or is it linked to permanent extrinsic stimulation (IL-4?, IL-21?, Antigen?), 2/do these cells express some new surface markers which were not detectable during our quite extensive B cell immunophenotyping? 3/is this stage linked to lupus susceptibility in these patients, or is it an indication for flare susceptibility? All these questions will have to be addressed in a new and large cohort of patients that will be longitudinally tested. Finally, the description of this subgroup of lupus patients adds some new insights on the different biological roads which can lead to a lupus phenotype.

## Supporting Information

Table S1 **Non normalized data of the 17 patients and 8 controls with probe identification, gene names and statistics.** List of genes with p-values less than 0.05 (Wilcoxon test).
(XLS)

Table S2 **List of differentially expressed gene with a FDR less than 0.01 in the 5 patients compared to controls.**
(XLS)
